# Photoelectrochemical Water Splitting by *SnO*_2_/CuO Thin Film Heterostructure-Based Photocatalysts for Hydrogen Generation

**DOI:** 10.3390/nano15221748

**Published:** 2025-11-20

**Authors:** Joun Ali Faraz, Tanvir Hussain, Muhammad Bilal, Khaleel Ahmad, Luminita-Ioana Cotirla

**Affiliations:** 1 Department of Physics, University of Management and Technology, Lahore 54770, Pakistan; joun.faraz@umt.edu.pk (J.A.F.);; 2 Center for Theoretical Physics, Khazar University, 41 Mehseti Str., Baku AZ1096, Azerbaijan; khalil7066616@gmail.com; 3 Department of Mathematics, Technical University of Cluj-Napoca, 400114 Cluj-Napoca, Romania; luminita.cotirla@math.utcluj.ro

**Keywords:** photocatalysis, heterostructure, green hydrogen, thermal evaporation, photocurrent density

## Abstract

The emission of greenhouse gases from fossil fuels creates devastating effects on Earth’s atmosphere. Therefore, a clean energy source is required to fulfill the energy demand. Hydrogen is considered an energy vector, and the production of green hydrogen is a promising approach. Photoelectrochemical (PEC) water splitting is the best approach to produced green hydrogen, but the efficiency is low. To produce hydrogen by PEC splitting water, semiconductor photocatalysts have received an enormous amount of academic research in recent years. A new class of co-catalysts based on transition metals has emerged as a powerful tool for reducing charge transfer barriers and enhancing photoelectrochemical (PEC) efficiency. In this study, copper oxide (CuO) and tin oxide ($SnO_{2}$) multilayer thin films were prepared by thermal evaporation to create an energy gradient between $SnO_{2}$ and CuO semiconductors for better charge transfer. To improve the crystallinity and reduce the defects, the prepared films were annealed in a tube furnace at 400 °C, 500 °C, and 600 °C in an argon inert gas environment. XRD results showed that $SnO_{2}$/CuO-600 °C exhibited strong peaks, indicating the transformation from amorphous to polycrystalline. SEM images showed the transformation of smooth dense film to a granular structure by annealing, which is better for charge transfer from electrode to electrolyte. Optical properties showed that the bandgap was decreased by annealing, which might be diffusion of Cu and Sn atoms at the interface. PEC results showed that the $SnO_{2}$/CuO-600 °C heterostructure exhibits the solar light-to-hydrogen (STH%) conversion efficiency of 0.25%.

## 1. Introduction

The world now strongly demands sustainable energy solutions because fossil fuel depletion, together with greenhouse gas emissions, damages our environment [1]. Solar energy and wind power, together with hydro generation, serve as sustainable power alternatives that are endlessly renewable. Solar energy represents a prominent choice because it exists widely, and it serves as a molecular basis for water splitting operations to produce hydrogen [2,3]. The energy carrier status of hydrogen presents an impressive opportunity to change existing global energy systems because it offers clean, renewable energy capabilities. The fuel serves as an attractive replacement for fossil fuels because of its concentrated energy content while releasing no greenhouse gases [4]. Photoelectrochemical (PEC) water splitting stands as one of the most efficient renewable methods among all hydrogen production techniques [5,6]. The water splitting reaction into hydrogen and oxygen takes place through solar irradiation by using semiconductor-based photocatalysts [5,7].

Advancements continue in hydrogen storage, together with transportation systems and use technologies, which point toward promising future development of hydrogen energy [8]. Governments across the globe spend money on hydrogen network development to establish a hydrogen-based economic system [9,10]. Green hydrogen production through water electrolysis operated by renewable energy sources will establish itself as a fundamental element for industry decarbonization and power generation and transportation systems decarbonization [11]. Researchers encounter obstacles in commercialization because of high production expenses, together with scarce efficient photocatalysts, along with stabilization issues. Research activities specifically work on finding affordable materials that boost hydrogen production speed and efficiency [12]. The breakthrough discovery of PEC water splitting with $TiO_{2}$ occurred when Faraji et al. (2019) introduced their method, which has initiated research to find different semiconductor materials [13]. The photoelectrochemical performance of $WO_{3}$, $Fe_{2}O_{3}$, and $Cu_{2}O$ transition metal oxides has been shown to improve through their abilities to absorb visible wavelengths and promote electrical charge flow [14]. $SnO_{2}$ shows that this material demonstrates both high electron mobility and stable performance, which makes it very suitable for use as a photoanode. The big bandgap prevents this semiconductor from absorbing visible light photons [15,16]. CuO shows achievement as a photocathode material in research because it effectively absorbs visible light through its narrow bandgap [17]. The investigations of semiconductor photocatalysts represent a key approach for advancing photoelectrochemical water splitting through research. The efficiency of the PEC water splitting process can be enhanced by modifying the properties of semiconductor photocatalysts, i.e, by creating energy gradient for better charge transfer for making heterostructures, having better change transfer between electrode and electrolyte by nanostructuring and enhance the solar light absorption by modulating the bandgap of the semiconductor [18,19,20].

Tin oxide ($SnO_{2}$) is an n-type semiconductor, has a large bandgap (3.6 eV), and shows remarkable chemical durability, but because of the large bandgap, most of the solar light passes through the material and is unable to produce electron-hole pairs [21]. CuO is a p-type semiconductor and has a narrow bandgap in the range of 1.2 eV to 1.6 eV [22]. Therefore, CuO is the best candidate for solar light absorption, but its valence band above the oxidation level of water becomes a bottleneck for the water splitting reaction [23].

Several studies have reported that combining n-type $SnO_{2}$ with p-type CuO forms a type-II heterojunction that enhances charge separation, suppresses recombination, and broadens light absorption into the visible range, thereby improving photocatalytic and photoelectrochemical (PEC) performance. Selleswari et al. (2019) demonstrated that $CuO-SnO_{2}$ heterostructures exhibit superior UV light-driven photocatalytic activity owing to efficient electron–hole separation across the interface [24]. Animasahun et al. (2021) synthesized multilayered $SnO_{2}$/CuO/$SnO_{2}$ thin films and observed improved photo absorption and reduced recombination losses compared with pristine oxides [25]. More recently, Salmanizadeh et al. (2024) reported that $SnO_{2}$-CuO heterostructured photocatalysts synthesized via a simple solution process showed enhanced electron mobility and visible-light-driven PEC efficiency [26]. However, most of these studies employed sol-gel or hydrothermal techniques, which often result in non-uniform interfaces and limited crystallinity.

In this study, $SnO_{2}/CuO$ thin-film-based heterostructures are formed by thermal evaporation and then annealed at 400 °C, 500 °C, and 600 °C. The formation of heterostructures will create an energy gradient, which helps to separate the generated charge carriers efferently, which will reduce the combination rate, and it will also resolve the issue of oxidation of water because the valence band edge of $SnO_{2}$ is below the oxidation level of water. The annealing will definitely remove the defects in the structures and improve the crystallinity, which enables further better charge separation and reduces the recombination rate. As a result, the photocurrent response and solar light-to-hydrogen efficiency will be enhanced.

## 2. Experimental Procedure

**Figure 1:**
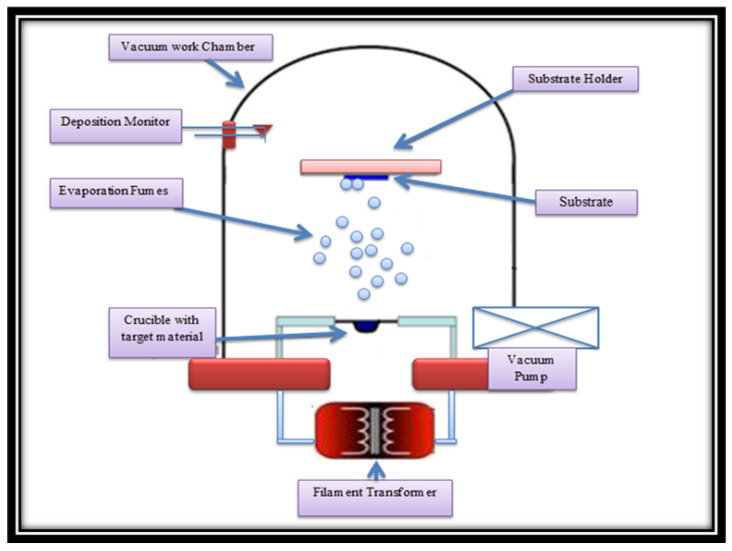
Schematic representation of $SnO_{2}$/CuO thin-film deposition via physical vapor deposition.

$SnO_{2}$/CuO heterostructures are formed by deposition of the CuO thin film followed by $SnO_{2}$ thin film by thermal evaporation. For this purpose, $SnO_{2}$ and CuO powders (Sigma Aldrich, St. Louis, MO, USA 99%>purity) were used as precursor materials. First of all, CuO powder of 1 g is placed in a tungsten boat in the thermal evaporation chamber, and the cleaned glass substrates and ITO substrates are placed in the substrate holder. When the vacuum of the chamber is $4\times 10^{-5}$ mbar, then 60 A current flows through the tungsten boat for resistive heating, and CuO powder is ablated and deposited on the glass substrates and ITO substrates. Similarly, then $SnO_{2}$ powder of 1 g is placed in a tungsten boat and again when the vacuum of the chamber is $4\times 10^{-5}$ mbar then 90 A current is passed through the tungsten boat to evaporate the $SnO_{2}$ powder. After 10 min of deposition, the $SnO_{2}$ film is deposited on the already-deposited CuO film; therefore, $SnO_{2}$/CuO heterostructures are formed. The deposition of CuO and $SnO_{2}$ thin films is illustrated in Figure 1.

**Figure 2:**
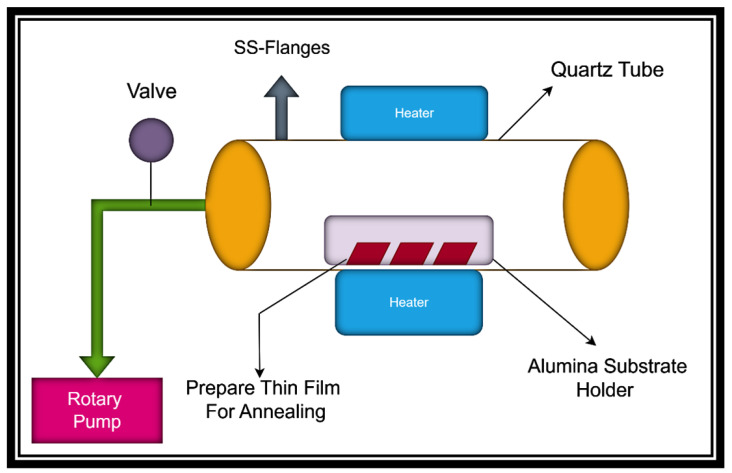
Schematic representation of $SnO_{2}$/CuO thin-film annealing via chemical vapor deposition.

After that, the prepared samples are annealed at 400 °C, 500 °C, and 600 °C in a tube furnace for 2 h in the presence of argon gas (99.9% purity) at a flow rate of 100 sccm. The temperature rise rate is set to 7 °C/min for heating and cooling. The annealing of $SnO_{2}$/CuO thin films in an argon environment is shown in Figure 2.

X-ray diffraction (XRD) analysis determined the structural properties of the synthesized thin films. A Bruker (AXS GmbH, Karlsruhe, Germany) D2-Phaser X-ray diffractometer was used for carrying out the measurements. XRD acquisition settings: Cu Kα radiation, 2θ range 20–80°, step size 0.02°. Scanning Electron Microscopy (SEM) was used to measure the morphological properties of the prepared sample. Optical properties were measured by spectrophotometry. Photoelectrochemical properties were measured by using a Gamry potentiostat (Gamry Instruments, Reference 3000, Warminster, PA, USA) setup in a three-electrode cell. The setup involves using a reference electrode made of Ag/AgCl, a counter electrode made of platinum wire, and a working electrode made of prepared samples. A concentration of 0.5 M $Na_{2}SO_{4}$ is used as the electrolyte. The linear sweep voltametry of samples was measured under dark and light conditions (100 mW.cm^−2^).

## 3. Results and Discussion

### 3.1. Structural Analysis

**Figure 3:**
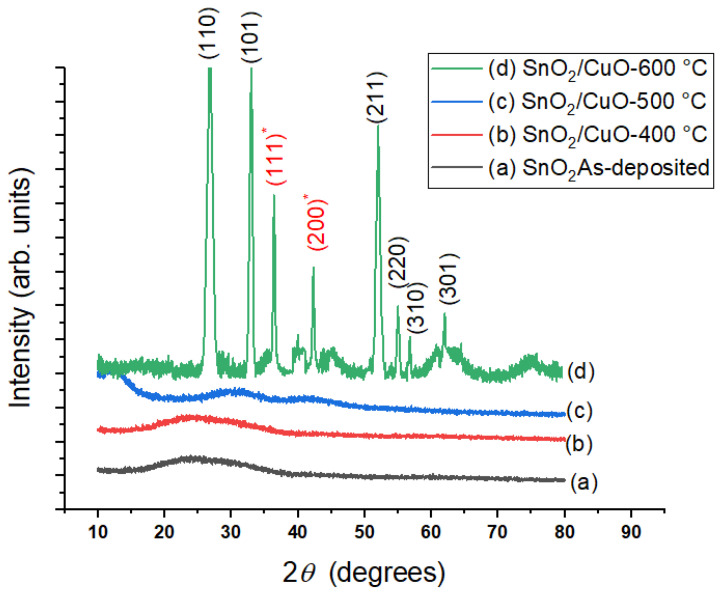
XRD patterns of (**a**) as-deposited $SnO_{2}$, (**b**) $SnO_{2}$/CuO-400 °C, (**c**) $SnO_{2}$/CuO-500 °C, and (**d**) $SnO_{2}$/CuO-600 °C thin films.

XRD patterns of as-deposited $SnO_{2}$ thin film and $SnO_{2}$/CuO-400 °C, $SnO_{2}$/CuO-500 °C, and $SnO_{2}$/CuO-600 °C thin films are shown in Figure 3. It was observed that as-deposited $SnO_{2}$ thin film, $SnO_{2}$/CuO-400 °C and $SnO_{2}$/CuO-500 °C are amorphous, while the $SnO_{2}$/CuO-600 °C thin film showed strong peaks, which indicates it is polycrystalline. It was noticed that peaks present at 2θ = 26.78°, 32.96°, 52.04°, 54.94°, 56.68°, 61.92° with corresponding reflection planes (110), (101), (211), (220), (310) of $SnO_{2}$ that are well matched with JCPDS card no. 41-1049 [27]. Similarly, peaks appeared at 2θ = 36.37°, 42.24° with the corresponding reflection planes (111)* and (200)* belonging to CuO. The peaks of CuO belong to a face-centered cubic crystal structure with the JCPDS card number 05-0667 [28]. The transformation from amorphous to crystalline indicates the arrangement of atoms and a decrease in defects, which enhances the charge carrier separation rate and decreases the recombination rates, which increases photocurrent response [29].

### 3.2. Morphological Analysis

**Figure 4:**
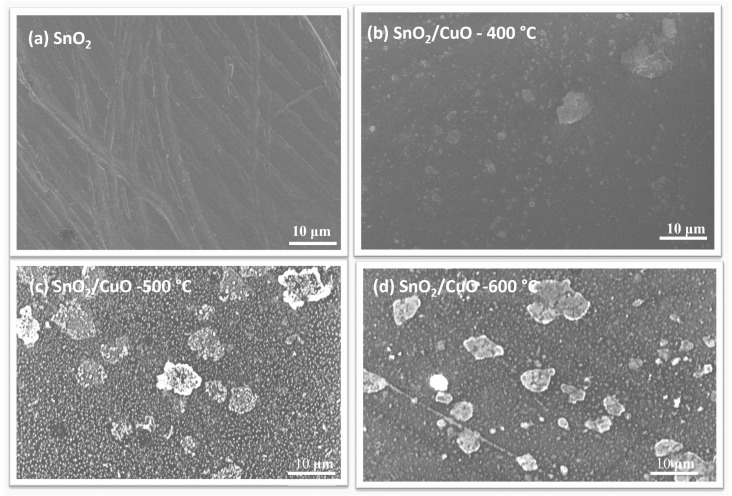
SEM images of (**a**) as-deposited $SnO_{2}$, (**b**) $SnO_{2}$/CuO-400 °C, (**c**) $SnO_{2}$/CuO-500 °C, and (**d**) $SnO_{2}$/CuO-600 °C.

Scanning Electron Microscopy (SEM) was used to analyze the surface morphology of $SnO_{2}$ and $SnO_{2}$/CuO thin films annealed at 400 °C, 500 °C, and 600 °C temperatures. Figure 4a is the as-deposited $SnO_{2}$ thin film, which reveals a smooth and dense surface with fine structural characteristics, indicating uniform and compact film formation. The $SnO_{2}$/CuO-400 °C thin film in Figure 4b exhibits tiny, distributed grains on the surface, indicating the start of development of the CuO phase and the establishment of a heterostructure interface. As the annealing temperature reached 500 °C Figure 4c, the film formed a granular shape with visible spherical and irregularly shaped particles, suggesting increased grain formation. At 600 °C, shown in Figure 4d, the $SnO_{2}$/CuO heterostructure showed bigger, well-defined grains with clear borders, indicating considerable grain development by thermal energy-driven diffusion. The formation of grains by increasing annealing temperature is suitable for charge transfer between the electrode and electrolyte resulting in an increase in photocurrent response [30].

### 3.3. Bandgap Determination

The optical properties of the prepared films were measured by a spectrophotometer. The spectrometry of the prepared samples is based on the Beer-Lambert principle

(1) $$I=I_{0}e^{-\alpha x}\;$$

**Figure 5:**
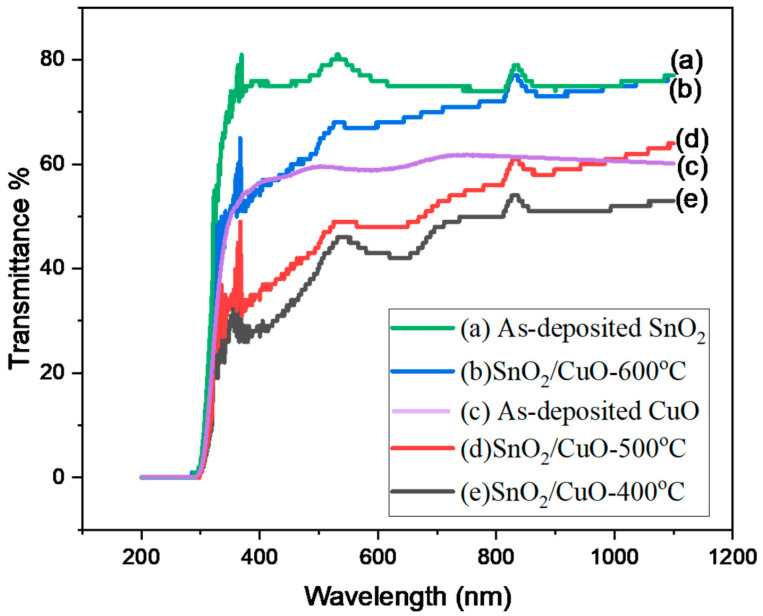
Transmittance spectra of (**a**) as-deposited $SnO_{2}$, (**b**) $SnO_{2}$/CuO-600 °C, (**c**) as deposited CuO (**d**) $SnO_{2}$/CuO-500 °C, and (**e**) $SnO_{2}$/CuO-400 °C.

Here, $I_{0}$ represents the incident light intensity, *I* represents the transmittance light intensity, α represents the absorption coefficient, and *x* represents the film thickness. The transmittance spectra of the prepared samples are shown in Figure 5.

Figure 5 represents the transmittance of $SnO_{2}$ and $SnO_{2}$/CuO thin film annealed at 400 °C, 500 °C, and 600 °C. It was noticed that all thin films are transparent in the visible region (380 to 760 nm). The transmittance of $SnO_{2}$/CuO-600 °C heterostructure thin film was increased by increasing the temperature. It is due to the decrease in defects and arrangements of atoms that allow light to pass through the sample [31]. The estimated average transmittance in the visible region is 75%. The following formula can be used to determine the absorption constant a.

(2) $$a=\frac{1}{d}ln\frac{1}{T_{f}}$$

**Figure 6:**
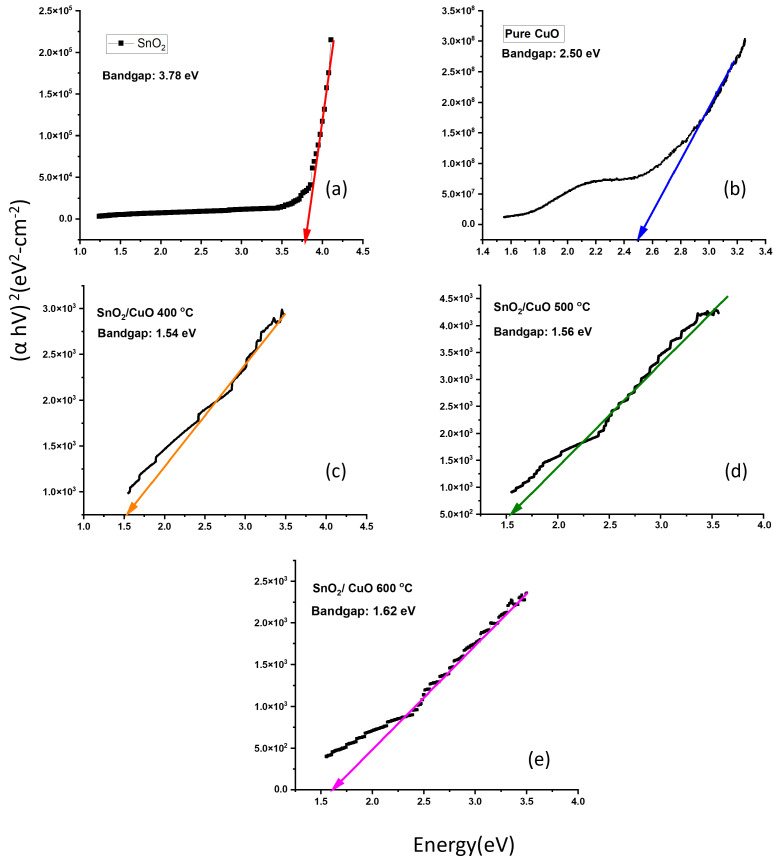
Tauc plot for as-deposited $SnO_{2}$ (**a**) as-deposited CuO (**b**) $SnO_{2}$/ CuO-400 °C (**c**) $SnO_{2}$/CuO-500 °C (**d**) $SnO_{2}$/CuO °C (**e**).

The thickness of the thin film is denoted by *d*, and the transmittance of the thin film is denoted by $T_{f}$. In the strong absorption region, the absorption coefficient is dependent on the incident photon energy (E). Tauc was the one who first proposed utilizing optical absorbance to determine the semiconductor bandgap ($E_{g}$). A Tauc plot method helped determine the optical band gap of all prepared thin films. The optical bandgap measurement of unprocessed $SnO_{2}$ thin film deposited onto Si (100) substrate presented a value of 3.78 eV (Figure 6a) that is well matched with the already reported values [32]. The as-deposited CuO thin film exhibited an optical band gap of 2.50 eV, as extracted from the Tauc-plot analysis (Figure 6b). The relatively high value of the band gap compared to typical values for bulk CuO suggests that the film is not purely CuO but rather contains a mixture of $Cu_{2}O$ and CuO phases. The presence of $Cu_{2}O$, (which has a higher bandgap than CuO) shifts the effective optical absorption edge upward, and thus the measured value of 2.50 eV is consistent with the literature for mixed $Cu_{2}O$/CuO phases [33]. The Tauc plot analysis revealed the bandgap values of $SnO_{2}$/CuO-400 °C as 1.54 eV, $SnO_{2}$/CuO-500 °C as 1.56 eV, and $SnO_{2}$/CuO-600 °C as 1.62 eV in that order. The reduction in the heterostructure thin films’ bandgap below pure $SnO_{2}$ and CuO occurs due to interface diffusion of Cu atoms and Sn atoms, which act as dopants and create dopant defect levels within the bandgap [34]. The $SnO_{2}$/CuO heterostructures fabricated by thermal evaporation in this work exhibit optical bandgap values between 1.54 eV and 1.62 eV, which are slightly lower than those reported for sol-gel or hydrothermally prepared heterostructures typically 1.65–1.80 eV [35]. This reduction indicates improved interfacial coupling and band alignment due to controlled annealing and film uniformity.

### 3.4. Photoelectrochemical Water Splitting

**Figure 7:**
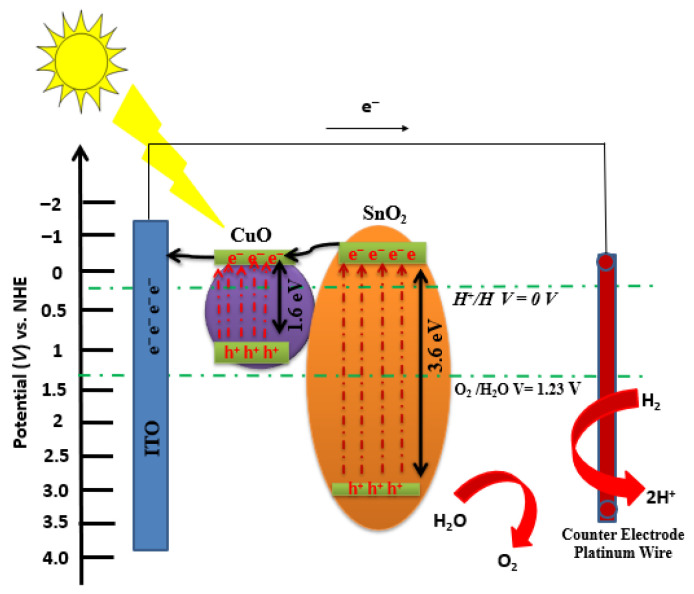
Plausible reaction mechanism of $SnO_{2}$/CuO heterostructure thin-film-based photocatalyst for H_2_ generation under solar light irradiation.

The photoelectrochemical properties of the as-deposited $SnO_{2}$, as-deposited CuO, $SnO_{2}$/CuO-400 °C, $SnO_{2}$/CuO-500 °C, and $SnO_{2}$/CuO-600 °C were measured by measuring the linear sweep voltammetry under dark conduction and under light conditions to estimate the photocurrent response. LSV results showed that the photocurrent density of pure tin oxide and copper oxide is small and was increased when $SnO_{2}$/CuO heterostructure is formed. It is due to the creation of an energy gradient that enhances the charge separation, and this phenomenon is illustrated in Figure 7, which explains the plausible reaction mechanism of oxidation and reduction reactions of water splitting.

It was also noticed that the photocurrent response was increasing in the $SnO_{2}$/CuO heterostructure by increasing the annealing temperature. The increase in photocurrent was due to the decrease in defects in the films, which results in a decrease in recombination rate and better charge separation and formation of granular structure by annealing, which if effective for better charge transfer between electrode and electrolyte results in an enhancement in reaction rate [36]. The solar light-to-hydrogen conversion efficiency (STH %) of the prepared samples was estimated according to the following Equation [19].

(3) $$STH\%=\frac{(1.23-V_{bias})\times J}{p}\times 100\%$$

where *P* is the intensity of the solar spectrum, *J* is the photocurrent density, and $V_{bias}$ is the applied voltage. Table 1 has been updated to include typical reference materials such as $TiO_{2}$, $WO_{3}$, and $Cu_{2}O$ for comparison. The obtained solar-to-hydrogen (STH %) conversion efficiencies of our thermally evaporated $SnO_{2}$/CuO thin films are very good relative to the literature reports on similar metal oxide heterostructures. In our study, the photocurrent density and STH % of the $SnO_{2}$/CuO heterostructures increased significantly compared with the pristine films. Although certain studies using sol-gel or hydrothermal routes have reported higher absolute STH values for more complex systems, the relative enhancement achieved in our work by forming a simple bilayer heterostructure is substantially larger. This clearly demonstrates the beneficial effect of controlled thermal evaporation, annealing crystallinity, and charge-transfer efficiency in $SnO_{2}$/CuO systems.

It was noticed that $SnO_{2}/CuO$-600 °C thin film showed STH% almost 1560 times more photocurrent response compared to pure CuO, which indicates that the formation of heterostructures enhances the hydrogen production and can be enhanced further by creating better-quality film and a better way to nanostructure them.

**Figure 8:**
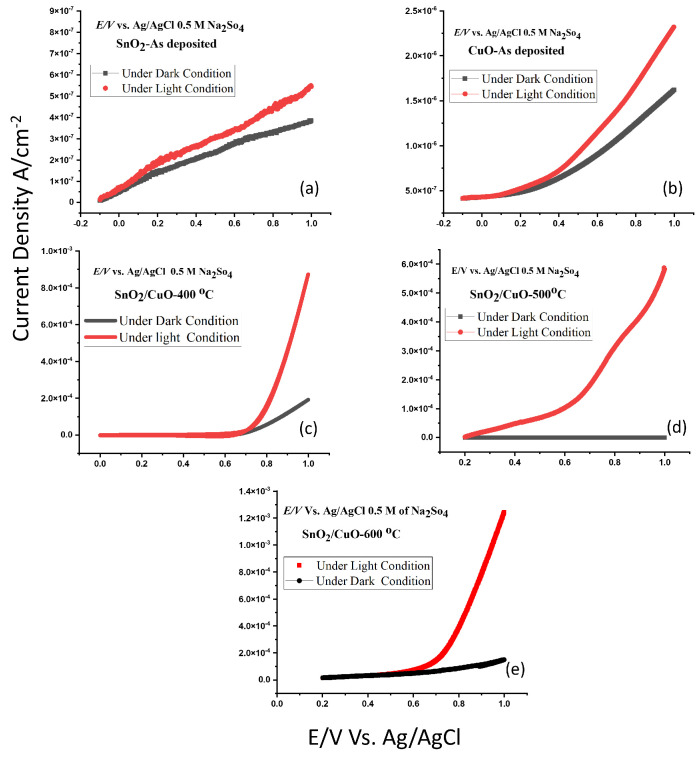
LSV graphs of pure $SnO_{2}$ (**a**), pure CuO (**b**), $SnO_{2}$/CuO-400 °C (**c**), $SnO_{2}$/CuO-500 °C (**d**), $SnO_{2}$ /CuO-600 °C, (**e**) thin films under dark and light conditions.

The value of photocurrent density under light conditions is where the value under dark conditions is obtained by applying a potential of 1 V (Figure 8). Similarly, the photocurrent response of $SnO_{2}/CuO$-400 °C, $SnO_{2}/CuO$-500 °C, $SnO_{2}/CuO$-600 °C is measured and given in Table 1. It was noticed that the photocurrent response of the heterostructure is more compared to the pure CuO and $SnO_{2}$ thin films. It is due to the creation of an energy gradient and the increased light absorption of the samples. The photocurrent response of the heterostructure annealed at 600 °C is more compared to $SnO_{2}/CuO$-400 °C and $SnO_{2}/CuO$-500 °C. It is due to the motion of $Cu_{2}O$ from CuO, which creates a better energy gradient for charge transformation. The $SnO_{2}/CuO$ heterostructures under 600 °C annealing temperature generated superior results in PEC measurements regarding their photocurrent density and hydrogen evolution efficiency. The photocurrent density obtained for the $SnO_{2}/CuO$–600 °C sample (0.25 STH %) betters many comparable systems reported previously, where STH efficiencies generally remain below 0.15% under similar conditions. These results confirm that the optimized thermal evaporation route enhances carrier separation, crystallinity, and PEC performance, underscoring the novelty of our deposition method.

**Figure 9:**
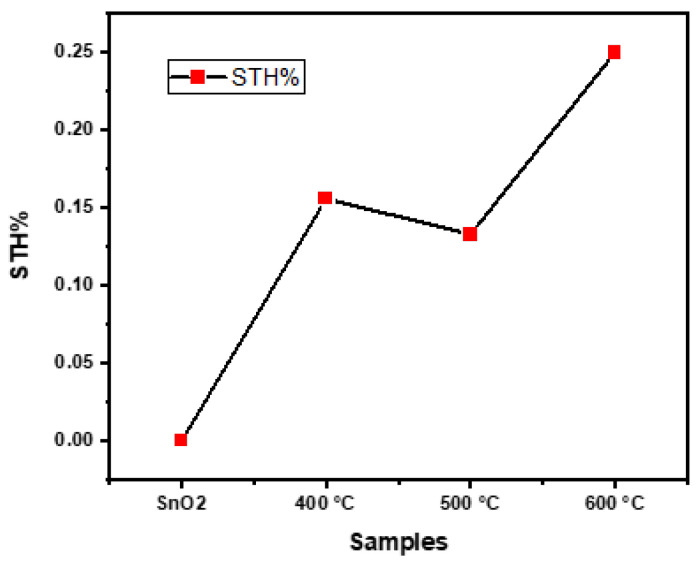
Solar-to-hydrogen conversion graph.

Figure 9 depicts a comparison of bare $SnO_{2}$, CuO, and $SnO_{2}$/CuO with a heterostructure.

## 4. Conclusions

The research investigation showed successful evidence that $SnO_{2}$/CuO thin-film heterostructures deliver efficient PEC hydrogen generation capabilities. An improved photocatalytic effect and advanced light absorption, together with increased charge separation, result from combining $SnO_{2}$ and CuO. The $SnO_{2}$/CuO heterostructures were prepared via a stepwise thermal evaporation process. The samples were annealed in an argon gas environment. Optical properties showed that pristine $SnO_{2}$ and CuO thin films showed good transmittance in the visible region, while heterostructure $SnO_{2}$/CuO thin films showed less transmittance in the visible region because of the increase in light scattering at the interface. XRD data depict that as-deposited $SnO_{2}$, heterostructure of $SnO_{2}$/CuO of 400 °C and 500 °C showed an amorphous phase due to the presence of defects and less planes for a present to generate peaks, while the heterostructure $SnO_{2}$/CuO films annealed at 600 °C are polycrystalline in nature, confirming improved grain growth and reduced lattice defects compared to lower-temperature samples. Tauc plots of the prepared samples showed that heterostructured $SnO_{2}$/CuO thin films have a smaller bandgap (1.56 eV) compared to pure $SnO_{2}$ (3.78 eV) and CuO (2.50 eV), which is attributed to the diffusion of CuO in the $SnO_{2}$ thin films, leading to enhanced light absorption and charge transfer. Photoelectrochemical properties showed that heterostructure $SnO_{2}$/CuO thin films showed better photocurrent response and solar-to-hydrogen conversion efficiency than pristine films. The highest efficiency 0.25% was achieved for $SnO_{2}$/CuO-600 °C, showing the beneficial impact of optimal annealing temperatures on film performance. Annealing and enhanced manufacturing procedures can help to further optimize hydrogen production. Future research will focus on improving thin-film stability and investigating other metal oxide combinations to enhance hydrogen generation efficiency. Furthermore, nanostructuring and enhanced deposition processes are predicted to provide smoother coatings with better photoelectrochemical properties.

## Figures and Tables

**Table 1:** Solar light-to-hydrogen conversion efficiencies of $SnO_{2}$, CuO, $SnO_{2}$/CuO-400 °C, $SnO_{2}$/CuO-500 °C, $SnO_{2}$/CuO-600 °C.

Samples Name	STH%	Reference
$TiO_{2}$(anatase)	0.02	Arora et al. (2023) [37]
$WO_{3}$	0.05	Arora et al. (2023) [37]
$Cu_{2}O$	0.10–0.15	Xiao et al. (2023) [38]
$SnO_{2}$	$3.70\times 10^{-5}$	This work
CuO	$1.60\times 10^{-4}$	This work
$SnO_{2}/CuO$ at 400 °C	$1.56\times 10^{-1}$	This work
$SnO_{2}/CuO$ at 500 °C	$1.33\times 10^{-1}$	This work
$SnO_{2}/CuO$ at 600 °C	$2.5\times 10^{-1}$	This work

## Data Availability

Dataset available on request from the authors.
